# Improving CMS SEP-1 Sepsis Bundle Compliance With Additional Documentation Measures

**DOI:** 10.51894/001c.144142

**Published:** 2025-09-30

**Authors:** Ahmed Nassem

**Affiliations:** 1 Department of Emergency Medicine McLaren Oakland

## 43

### INTRODUCTION/BACKGROUND

The Centers for Medicare and Medicaid Services (CMS) instituted the SEP-1 initiative, designed to improve sepsis identification and treatment.1 2 To implement this initiative at a community teaching hospital, a multi-phase Plan-Do-Study-Act (PDSA) Quality Improvement-Patient Safety (QI-PS) project was initiated. All phases received IRB approval.

### AIMS/OBJECTIVES

The project’s goal was to increase SEP-1 protocol compliance to 69% or greater. Baseline data, collected from November 2020 to April 2021, indicated an average compliance of 57%.3

Phase 1 involved the creation of a sepsis macro and order set in the electronic medical record. Phase 2 introduced the used of monthly education sheets for first-year emergency-medicine and off-service residents. Since neither of these interventions met the target compliance rate, Phase 3, the required completion of a paper Sepsis Sheet, was implemented.

### METHODS

The Phase 3 team designed a Sepsis Sheet for any patient presenting with sepsis symptoms in the Emergency Department. This paper form provided a means of documenting the time of sepsis diagnosis, patient’s ideal body weight, patient’s actual body weight, and time stamps for drawing of lactic acid, blood cultures, completion of IV fluid bolus, starting of vasopressors, and reassessment times. Staff were trained on use of the sheet during monthly meetings. The Sepsis Sheet was required to be completed by the time the patient was admitted to an inpatient hospital unit. Data from three months before and after the implementation of the Sepsis Sheet (April 2024) were evaluated.

### RESULTS

Utilizing the Sepsis Sheet, SEP-1 compliance increased from 54% to 77% (p=0.036). There was no significant change in length of hospital stay (days) (p=0.673).

### DISCUSSION

The addition of the Sepsis Sheet and its required completion proved to be critical toward improving SEP-1 protocol compliance.

